# TSLP bronchoalveolar lavage levels at baseline are linked to clinical disease severity and reduced lung function in children with asthma

**DOI:** 10.3389/fped.2022.971073

**Published:** 2022-09-29

**Authors:** Elizabeth Chorvinsky, Gustavo Nino, Kyle Salka, Susana Gaviria, Maria J. Gutierrez, Dinesh K. Pillai

**Affiliations:** ^1^Division of Pediatric Pulmonary and Sleep Medicine, Children's National Medical Center, George Washington University, Washington, DC, United States; ^2^Division of Pediatric Allergy, Immunology and Rheumatology, Johns Hopkins University, Baltimore, MD, United States

**Keywords:** TSLP, asthma, children, lung, BAL (bronchoalveolar lavage)

## Abstract

**Rationale:**

Thymic stromal lymphopoietin (TSLP) is increasingly recognized as a key molecule in asthma pathogenesis and as a promising therapeutic target in adults. In contrast, in asthmatic children the clinical relevance of TSLP secretion in the lower airways has been remarkably understudied. We tested the hypothesis that pulmonary TSLP levels in asthmatic children correlate with clinical severity, airway inflammation and lower airway obstruction.

**Methods:**

Bronchoalveolar lavage (BAL) samples and relevant clinical data were collected from asthmatic children undergoing clinically indicated bronchoscopy at Children's National Hospital in Washington D.C. Protein levels of TSLP, IL-5, IL-1β, and IL-33 were quantified in BAL at baseline and correlated with individual severity and clinical features including spirometry, serum IgE and eosinophils, BAL neutrophil and eosinophil counts.

**Results:**

We enrolled a total of 35 asthmatic children (median age: 9 years). Pediatric subjects with severe asthma had greater TSLP BAL levels at baseline relative to mild or moderate asthmatic subjects (*p* = 0.016). Asthmatic children with the highest TSLP levels (>75th percentile) had higher IL-5 and IL-1β BAL levels and greater lower airway obstruction (lower FEV1/FVC ratios).

**Conclusion:**

Our study demonstrates for the first time that higher pulmonary TSLP levels obtained at baseline are linked to asthma disease severity in a subset of children. These data indicate that TSLP may play a key role in the pathogenesis of pediatric asthma and thus provide initial support to investigate the potential use of anti-TSLP biologics to treat severe uncontrolled asthmatic children.

## Introduction

Asthma is the most common chronic lung disease of childhood and accounts for major healthcare utilization and economic burden worldwide (1). Despite advances in treatment, awareness and education, management of pediatric asthma continues to be a challenge for health care providers, particularly in the most severe forms of the disease (2). Severe asthma in children is associated with significant morbidity and mortality as well as lack of response to standard therapies (3). There is a critical need to conduct studies to understand the pathogenesis of severe asthma in children to guide emergent therapies that specifically target molecular drivers of airway inflammation and hyperreactivity.

Studies using airway specimens derived from adults with asthma have confirmed that type 2 pro-asthmatic cytokines, such as Thymic Stromal Lymphopoietin (TSLP), are key mediators in the pathogenesis of the disease (4). TSLP is a primarily epithelial-derived cytokine released in response to environmental stimuli such as respiratory viruses and allergens (5). Mechanistic studies indicate that TSLP drives eosinophilic inflammation and structural changes of the airway in asthma through actions on a wide variety of adaptive and innate immune cells and structural cells (5). This fundamental knowledge has led to the discovery of novel anti-TSLP asthma therapies (Tezepelumab) for adults (6). In a phase 2, randomized, double-blind, placebo-controlled trial, Tezepelumab reduced asthma exacerbations by up to 71% compared with placebo in patients with severe, uncontrolled asthma across the spectrum of inflammatory phenotypes, and improved lung function and asthma control (7). Phase 3 trials have similarly demonstrated a reduction in exacerbations as well as improved lung function in participants who received Tezepelumab (6). In contrast, little is known about the clinical relevance of TSLP secretion in the lower airways of children with asthma. As lung specimens are not easily available in the pediatric population, there is limited understanding of the immunobiology of the lower airways of infants, children and adolescents despite compelling evidence that asthma often begins in early life (8).

Our team has previously reported that significant airway secretion of TSLP occurs during viral respiratory infections in young children particularly in those who have recurrent wheezing illnesses or have been diagnosed with asthma (9). These findings concur with several studies in animal models demonstrating that, during early life, TSLP is an essential component of the cascade of immune pro-asthmatic mediators during exposure to allergens and viruses (10–12). Malinczak et al. identified that in addition to promoting altered lung immune responses during neonatal RSV-infection, TSLP leads to long-term alterations including Th2 polarization and enhanced airway reactivity later in life (10, 13). These mechanistic findings in animal models suggest that TSLP may be a key therapeutic target early in life (13). While our human-based studies have confirmed that TSLP is produced *in-vivo* in the nasal airway of children during acute exacerbations (9, 14), it is not feasible to conduct similar *in-vivo* assessments of the lower airways via bronchoscopy during acute illnesses due to the risk and ethical concerns. Thus, it is presently unknown if TSLP is a key molecular mediator in the lungs of asthmatic children as previously established in adult asthmatics. Addressing this gap is critical as anti-TSLP and other biologics targeting type 2 inflammation represent a promising future alternative to reduce morbidity and mortality in children with severe uncontrolled asthma.

The overarching goal of this study was to test the hypothesis that pulmonary TSLP levels in children with asthma correlate with clinical severity, companion airway inflammation and lower airway obstruction. For this purpose, we obtained and analyzed bronchoalveolar lavage specimens collected via bronchoscopy in a population of well-characterized asthmatic children. We defined companion cytokine profiles and clinical characteristics of asthmatic children with the highest TSLP levels (>75th percentile). The impact of this work is that it demonstrates for the first time that TSLP levels in the lungs of children with asthma are linked to clinical severity and thus provides needed support to investigate the potential use of anti-TSLP or other biologics targeting type 2 inflammation in the pediatric population.

## Methods

### Study design

In this single-center, observational study we included children and adolescents (aged 1–18 years) clinically diagnosed with asthma undergoing bronchoscopy as part of evaluation in our asthma program at Children's National Hospital (CNH) in Washington, DC. We only included subjects with available bronchoalveolar lavage specimens obtained for clinical purposes in CNH. All individuals had bronchoscopy at baseline and we specifically excluded cases in which BAL was obtained emergently during acute respiratory illnesses. All clinical and demographic variables were obtained by parental interview or by reviewing EMR. The Institutional Review Board (IRB) of Children's National Medical Center, Washington D.C. approved the study and informed parental written consent was obtained in all research subjects included.

### Bronchoalveolar lavage collection and cytokine measurements

Pulmonary airway secretions were collected for clinical purposes using a standard BAL technique by wedging a bronchoscope in 2nd generation bronchi or further down. Gentle suctioning was used to instill and retrieve up to 2 mL/kg normal saline in each individual. Collected BAL fluid was immediately centrifuged at 1,000 g for 5 min to remove debris. Supernatant fluid was aliquoted and stored at −80°C until further analysis. A portion of BAL fluid was analyzed for protein levels of TSLP, IL-5, IL-1β, and IL-33 with a commercially available ultrasensitive electrochemiluminescence assays (MesoScale Discovery, MSD, Rockville, MD) using MSD standards and quality controls. Quality of assayed samples was examined in the Discovery Workbench software. Samples with values below the lower limit of detection (LLD) according to manufacturers' instructions were assigned the sqrt2/LLD as previously described (9, 14).

### Clinical variables

As previously described (15, 16), asthma was defined using National Asthma Education and Prevention Program (NAEPP) guidelines for the diagnosis, classification, and management of asthma in children, which stratify asthma severity based on clinical impairment (daytime and nighttime symptoms, activity limitation, and lung function testing) (17). Clinical classification of asthma severity was conducted independently and blinded to cytokine data analyses. Severe asthma is defined as “asthma that requires Step 4 or 5 treatment, e.g., high dose ICS-LABA (ICS: inhaled corticosteroid, LABA: long-acting beta agonist), to prevent it from becoming "uncontrolled', or asthma that remains ‘uncontrolled' despite this treatment” (18). Per NAEPP's Guidelines (17); Step 4 for Children aged 0–4 years consists of either daily medium dose ICS-LABA plus PRN SABA (short-acting beta agonist) OR daily medium dose ICS plus Montelukast and PRN (PRN: as needed) SABA (short-acting beta agonist). Step 5 consists of daily high-dose ICS-LABA plus PRN SABA and daily high-dose ICS plus Montelukast and PRN SABA. For ages 5–11 years, Step 4 consists of daily and PRN combination medium-dose ICS-formoterol with the alternatives of daily medium-dose ICS-LABA and PRN SABA or daily medium-dose ICS + LTRA (Leukotriene receptor antagonist) OR daily medium-dose ICS plus Theophylline and PRN SABA. Step 5 for this age group preferably consists of daily high-dose ICS-LABA and PRN SABA OR alternatively: daily high-dose ICS plus LTRA or daily high-dose ICS plus Theophylline, and PRN SABA. Clinical variables collected in the study included the following: date of BAL collection, age, gender (male, female), race/ethnicity (white, black, Hispanic), BMI percentile, Serum and BAL eosinophils, serum and BAL neutrophils, FeNO, Serum IgE levels, spirometry, asthma severity and step level, as well as having a history of PICU admission.

### Statistical analysis

Differences between continuous variables were analyzed using non-parametric Mann-Whitney *U* test. Associations between categorical variables were analyzed using the *X*^2^ test. All statistical tests were two-tailed, and the significance level used was *p* < 0.05. The data were analyzed with the Minitab V.19.1.

## Results

### Study subjects and clinical features according to asthma severity

We included a total of 35 children with asthma in this study. The median age was 9 years (IQR 5.7). Of these subjects, 71.4% were male and 57.1% were African American. To examine different clinical parameters and cytokine responses according to asthma severity categories, we divided all the study subjects into two groups based on NAEPP guidelines. In one group we included individuals that were mild or moderate in asthma severity (*n* = 11) and in the other group we included those who were considered severe asthma (*n* = 24). A complete comparison of demographic and clinical variables between these two groups is presented in Table 1.

**Table 1:** Demographics and clinical characteristics according to asthma severity.

	**Total**	**Asthma mild/moderate**	**Asthma severe**	***P* ^†^**
Number of subjects	35	11	24	
Age at enrollment, median years	9 (4.2)	8.6 (3.3)	9 (4.68)	0.558
Sex, male % (*n*)	71.4 (25)	81.8 (9)	66.7 (16)	0.36
African American % (*n*)	57.1 (20)	63.6 (7)	83.3 (20)	0.198
Caucasian, % (*n*)	11.4 (4)	9 (1)	12.5 (3)	0.769
>85%ile BMI %(median)	84 (58.3)	74 (55.45)	91.1 (65.9)	0.543
Eczema, % (*n*)	48.6 (17)	36.4 (4)	54.2 (13)	0.328
Food allergies % (*n*)	31.4 (11)	36.4 (4)	29.2 (7)	0.670
Allergic rhinitis, % (*n*)	82.9 (29)	81.8 (9)	83.3 (20)	0.912
Elevated serum Eosinophils (>0.3), % (*n*)**	60 (21)	27.3 (3)	75 (18)	0.008
Serum Eosinophils, median K/mcL (IQR)*	0.68 (0.5)	0.115 (0.52)	0.49 (0.38)	0.036
Serum Neutrophils, median K/mcL (IQR)	4.5 (2.4)	2.72 (2.74)	3.51 (2.25)	0.638
Serum IgE, median IU/mL (IQR)	417 (780)	209 (802)	503 (715)	0.176
Elevated serum IgE (>300), % (*n*)	60 (21)	27.3 (3)	75 (18)	0.008
FeNO, median ppb (IQR)	59 (43)	13 (11)	43.5 (38.88)	0.006
BAL % Eosinophils, median % (IQR)	13 (13)	0 (7)	3.5 (13)	0.082
BAL % neutrophils, median % (IQR)	64 (54)	23 (51)	37 (55.75)	0.286
PICU Admit ever, % (*n*)	25.7 (9)	9 (1)	33.3 (8)	0.112
Step Level 4 or 5, % (*n*)	88.6 (31)	63.6 (7)	100 (24)	0.002
FEV1, % Predicted, median values (IQR)	86.5 (18.5)	95 (26)	83 (15)	0.117
FEV/FVC, ratio reported as %(IQR)	84 (16.75)	88 (12)	77 (18)	0.007
FEF25-75, % predicted (IQR)**	65.5 (41)	96 (35)	58 (37)	0.006

^*^Numeric data expressed as median and interquartile range (IQR).

^†^P < 0.05 considered significant for each pairwise comparison using Mann-Whitney non-parametric test for continuous variables and by the chi-square test for categorical variables.

We confirmed the severe asthma severity status as per NAEPP's Guidelines (17) using multiple clinical parameters and lung function (Table 1). Relative to the group with mild/moderate asthma, children with severe asthma had significantly decreased FEV 1% predictive, decreased FEV/FVC ratios and decreased FEF 25–75% predicted (Table 1). The group of severe asthmatic children also had higher probability of using more medication for asthma according to NAEPP guidelines (Step level 4 or 5 as defined in methods, Table 1). In addition, we found that individuals in the severe asthma category were more likely to exhibit atopic features including higher serum eosinophils and IgE values (Table 1).

### TSLP and companion cytokine bronchoalveolar profiles based on asthma severity

**Figure 1:**
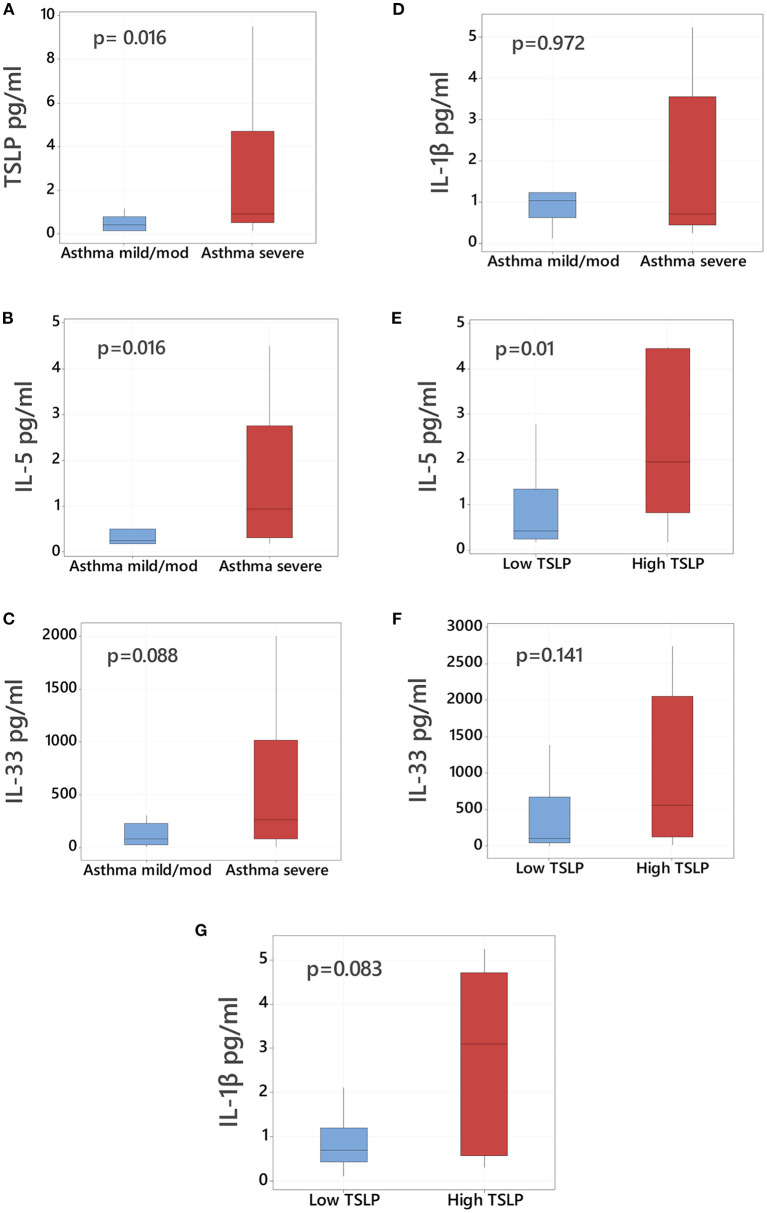
TSLP bronchoalveolar levels in asthmatic children according to severity. Comparison of bronchoalveolar lavage (BAL) protein levels of **(A)** TSLP, **(B)** IL-5, **(C)** IL-33, and **(D)** IL-1β in mild/moderate asthmatic children (left blue boxplot, *n* = 11) vs. severe asthmatic children (right red boxplot, *n* = 24). Comparison of BAL levels of **(E)** IL-5, **(F)** IL-33, and **(G)** IL-1β in asthmatic children with high TSLP (≥75th percentile) vs. low TSLP (<75th percentile) BAL levels. Data presented as boxplots representing 25th−75th percentiles.

We first examined baseline bronchoalveolar lavage (BAL) cytokine profiles according to asthma severity. As shown in Figure 1, we found the severe asthma cohort had overall increased levels of TSLP (median values, pg/mL, 0.409 vs. 0.923, *p* = 0.016, Figure 1A) and IL-5 (median values, pg/mL, 0.248 vs. 0.940, *p* = 0.016, Figure 1B) compared to the mild/moderate asthma group. Severe asthma also had higher levels of IL-33, though these differences did not reach statistical significance (median values, pg/mL, 78.88 vs. 262.14, *p* = 0.088, Figure 1C). We did not identify significant differences between mild/moderate vs. severe asthma cohorts in BAL protein levels of the pro-inflammatory cytokine IL-1β (Figure 1D).

We next examined the companion cytokine profiles in children with the highest TSLP levels. Subjects were considered to have high TSLP if the measured levels in their BAL were >75th percentile (2.24 pg/mL). All children with high TSLP BAL levels (*n* = 9) were classified as severe asthma and had significantly elevated levels of IL-5 (median values in pg/mL, 0.403 vs. 1.95, *p* = 0.01, Figure 1E). Subjects with high TSLP also demonstrated elevated IL-1β and IL-33, although these differences were not statistically significant (Figures 1F,G). High TSLP BAL levels were not associated with higher serum IgE levels (median values IgE 410 IU/mL in high TSLP vs. 417 IU/mL in low TSLP, *p* = 0.752, Table 2).

**Table 2:** Clinical features according to high airway TSLP status in BAL.

	**Total**	**Low TSLP**	**High TSLP**	***P* ^†^**
Number of subjects	35	26	9	
Age at enrollment, median years	9 (4.2)	8.85 (3.48)	12 (9.55)	0.213
Sex, male % (*n*)	71.4 (25)	76.9 (20)	55.6 (5)	0.221
African American % (*n*)	57.1 (20)	76.9 (20)	77.8 (7)	0.958
Caucasian, % (*n*)	11.4 (4)	7.7 (2)	22.2 (2)	0.238
>85%ile BMI % (median)	84 (58.3)	80.9 (57.5)	93.5 (60.5)	0.338
Eczema, % (*n*)	48.6 (17)	50 (13)	44.4 (4)	0.774
Food allergies, % (*n*)	31.4 (11)	34.6 (9)	22.2 (2)	0.490
Allergic rhinitis, % (*n*)	82.9 (29)	88.5 (23)	66.7 (6)	0.135
Elevated serum Eosinophils (>0.3), % (*n*)**	60 (21)	62.5 (15)	66.7 (6)	0.825
Serum Eosinophils, median K/mcL (IQR)	0.68 (0.5)	0.49 (0.57)	0.38 (0.37)	0.984
Serum Neutrophils, median K/mcL (IQR)	4.5 (2.4)	3.08 (2.28)	3.63 (3.04)	0.824
Serum IgE, median IU/mL (IQR)	417 (780)	417 (781)	410 (862)	0.752
Elevated serum IgE (>300), % (*n*)	60 (21)	54.2 (13)	62.5 (5)	0.768
FeNO, median ppb (IQR)	59 (43)	29 (49.8)	31.25 (34.8)	0.792
BAL % Eosinophils, median % (IQR)	13 (13)	1 (7.25)	4 (33)	0.439
BAL % Neutrophils, median % (IQR)	64 (54)	25 (52.5)	39 (67.5)	0.365
PICU Admit ever, % (*n*)	25.7 (9)	26.9 (7)	22.2 (2)	0.914
Step level 4 or 5, % (*n*)	88.6 (31)	84.6 (22)	100 (9)	0.211

^*^Numeric data expressed as median and interquartile range (IQR).

^†^P < 0.05 considered significant for each pairwise comparison using Mann-Whitney non-parametric test for continuous variables and by the chi-square test for categorical variables.

### Asthmatic children with high pulmonary TSLP levels have lower lung function

**Figure 2:**
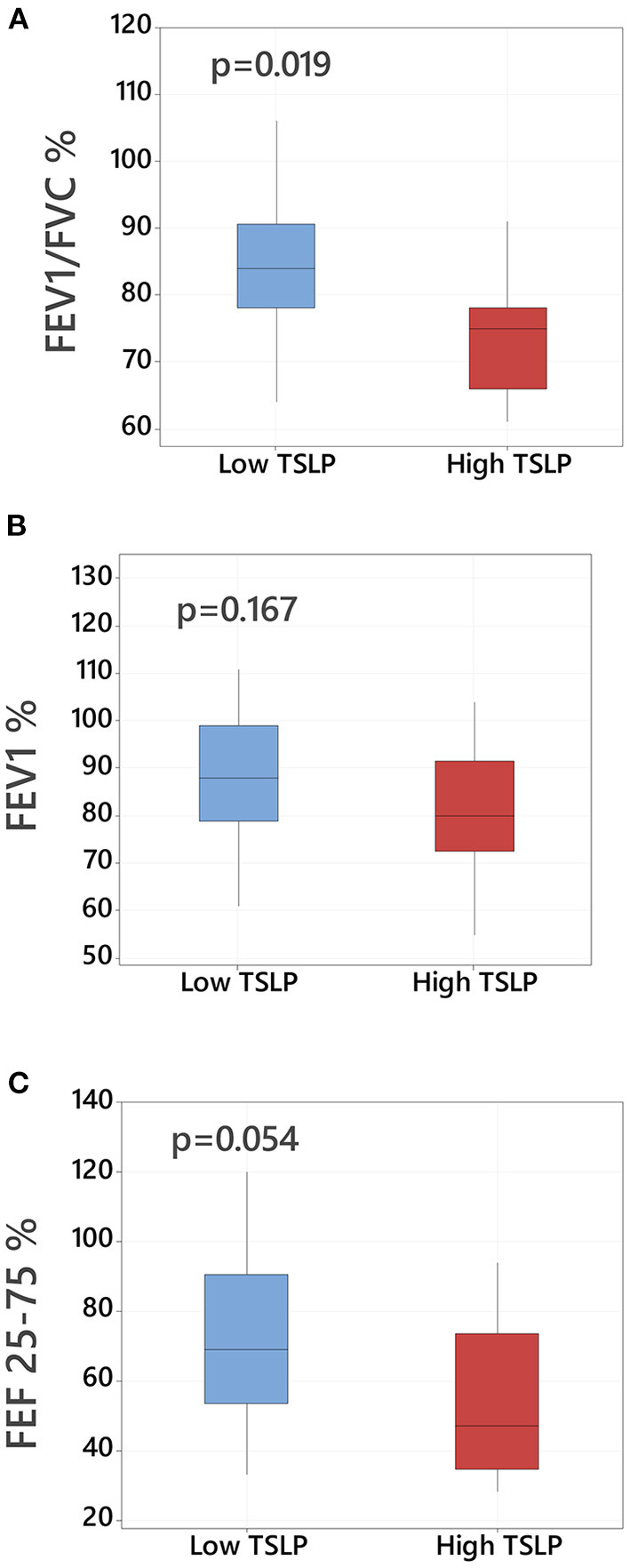
Lower airway obstruction and baseline TSLP pulmonary levels in asthmatic children. Comparison of lung function parameters in asthmatic children with high TSLP (≥75th percentile) vs. low TSLP (<75th percentile) BAL levels including **(A)** FEV1/FVC % predicted, **(B)** FEV1 % predicted and **(C)** FEF 25–75/% predicted, Data presented as boxplots representing 25th−75th percentiles.

We compared lung function in children with asthma based on presence of high pulmonary TSLP levels >75th percentile. Subjects with high TSLP levels demonstrated greater degree of lower airway obstruction based on lower FEV/FVC ratios (reported as a % predicted, 84 vs. 75, *p* = 0.019, Figure 2). Children with high TSLP also demonstrated decreased FEV 1% and decreased FEF 25–75% predicted although these differences did not reach statistical significance (Figure 2). There were no other significant differences in demographic or clinical characteristics between high and low TSLP groups. A full comparison of demographics and clinical variable comparisons between asthmatic children with and without high pulmonary TSLP levels is presented in Table 2.

## Discussion

Our results are the first to demonstrate that, during baseline bronchoscopic evaluation, the most severe pediatric asthma cases have higher TSLP protein levels in the lungs. Similar findings have been reported in the adult population (13), however, there is paucity of studies reporting BAL cytokine data in children with severe asthma. The main findings of our study are that (1) higher TSLP levels are associated with increased asthma disease severity in children, and (2) children with high TSLP levels (>75th percentile) are characterized by having increased pulmonary IL-5 levels together with greater airway obstruction (FEV1/FVC % predicted) but without other characteristic phenotypical features. These data suggest there is a subset of children with asthma that may have a lower airway inflammatory component linked to high TSLP levels and thus may benefit from emerging targeted biologic therapies against this master type 2 cytokine (19).

TSLP is a primarily epithelial-derived cytokine, released in response to environmental stimuli such as respiratory viruses and allergens (20). Cell based and animal model studies have implicated TSLP in allergic diseases, such as asthma, highlighting its key role in allergic type 2 responses in the airways and the TH2 polarization of the immune response to an allergic phenotype (20, 21). Murine models have provided further evidence for the role of TSLP in airway inflammation and asthma. Lack of TSLP expression via gene knock-out in animal models has demonstrated a protective effect against allergic inflammation, while inhibition of TSLP also results in reduced airway remodeling (22–24). TSLP's involvement in asthma also appears to extend beyond TH2 polarization of the type of response. Animal studies have implicated TSLP as having a crucial role in airway remodeling and steroid insensitivity of resident airway immune cells. Inhibition of TSLP has also demonstrated reduced remodeling of the airways in mouse models (25, 26). Targeting TSLP in the airways of mice prior to OVA allergic sensitization reduces airway inflammation, suggesting that TSLP is a promising therapeutic target for allergic asthma (27).

TSLP has similarly impactful effects in human airways and allergic immune responses. When stimulated with a viral mimic, bronchial epithelial cells from asthmatics secrete disproportionately high levels of TSLP compared to healthy subjects (28). As with animal models, increased TSLP is linked to airway remodeling in human airway samples (23). Adult asthmatic subjects demonstrate significantly elevated TSLP levels in the airways (19), and decreased lung function has been associated with elevated airway TSLP levels in the adult population (29). As a result, TSLP is now considered a promising biomarker and therapeutic target to improve outcomes for adults with uncontrolled severe asthma (19, 30).

While it is clear that TSLP plays a crucial role in asthma pathogenesis in adults, there is still a critical need to elucidate the role of this molecule in the pediatric population. Understanding how TSLP secretion is related to asthma severity and type 2 lower immune responses in children is needed to develop new targeted treatments for this age group. Our prior studies have shown that TSLP is the primary type 2 airway epithelial response of human infants during viral infections (14, 31), which is highly relevant as early-life viral infections predispose to subsequent asthma development later in life (32). In our current study we found that children with severe asthma have higher TSLP levels. The group of children with the highest TSLP levels (>75th percentile) also had increased BAL levels of IL-5 and IL-1β. The association of high TSLP levels with IL-5 is in agreement with extensive evidence linking TSLP with eosinophilic airway inflammation (33). Interestingly, several studies have also linked IL-1β to enhanced TSLP production (14, 34). We recently demonstrated *in-vitro* and *in-vivo* that IL-1β elicits TSLP secretion in the airways of young children (14). The positive effect of IL-1β in the induction of TSLP is seen in the epithelium as well as in immune cells including group 2 innate lymphoid cells (14). In our current study we also found that children with asthma and high pulmonary TSLP levels have greater lower airway obstruction (reduced FEV1/FVC ratios). Decreased lung function has been associated with elevated airway TSLP levels in the adult population (29) but to our knowledge this has not been reported in children. Collectively these novel molecular and clinical data indicate that TSLP may play a role in the pathogenesis of pediatric asthma, including lower airway inflammatory and obstructive features characteristic of the disease. Future studies are needed to further characterize pediatric severe asthma based on high TSLP levels to define best potential candidates for emerging anti-TSLP targeted therapies.

Although the main limitation of our study is the small sample size, we included a relatively large number of pediatric subjects considering the unique nature of the specimens (lower airway samples in children with asthma). We were able to test our main hypothesis (differences in baseline pulmonary TSLP levels according to asthma severity in children), but it is possible that our sample size prevented us from identifying additional differences in clinical parameters or airway cytokines among our study groups. We could not include comparisons in healthy subjects because all pediatric bronchoscopies had a clinical indication and research bronchoscopies cannot be performed in children. The TSLP values used here to define “High TSLP levels” are only pertinent to our study group and cannot be extrapolated to other settings with potential differences in BAL retrieval and analyses, including adults with asthma. It is also important to emphasize that the study was conducted in a specialized, tertiary referral hospital, thus the patients included may represent the extreme of the spectrum of severity of pediatric asthma, which may limit the generalization of results to other contexts. On the other hand, the key strength of our study is that these samples are from a unique pediatric population, which is typically difficult to assess in a research context, providing novel insights into a critically understudied age group. Of importance is that these BAL samples were not collected during an asthma exacerbation, demonstrating that baseline bronchoscopic assessment of the lower airways in asthmatic children may provide clinically relevant information about individual mechanisms of disease, outcomes and potential response to targeted therapy.

In summary, our study demonstrates for the first time that lung TSLP levels at baseline are associated with asthma severity in the pediatric population. Given that pediatric asthma is major health problem worldwide, we feel larger longitudinal studies are urgently needed to define additional features of TSLP production in the lungs of asthmatic children. This may include biomarkers, endotypes, and phenotypes to select best candidates for the emergent TSLP-driven asthma therapies addressing the precise underlying airway pathogenesis and lung immunobiology.

## Data availability statement

The data that support the findings of this study are available on reasonable request from the corresponding author, GN. The data are not publicly available due to their containing information that could compromise privacy of research participants.

## Ethics statement

Approval for human subject research was granted by the Institutional Review Board of Children's National Hospital in Washington, DC. Written informed consent from the participants' legal guardian/next of kin was not required to participate in this study in accordance with the national legislation and the institutional requirements.

## Author contributions

EC, GN, and DP: study design, data collection, and analysis. All authors: manuscript drafting, editing, and approval.

## Funding

Partially funded by NIH Grants R01HL141237 (GN), K23HD104933 (MG), and 1U01EB021986 (DP).

## Conflict of interest

The authors declare that the research was conducted in the absence of any commercial or financial relationships that could be construed as a potential conflict of interest.

## Publisher's note

All claims expressed in this article are solely those of the authors and do not necessarily represent those of their affiliated organizations, or those of the publisher, the editors and the reviewers. Any product that may be evaluated in this article, or claim that may be made by its manufacturer, is not guaranteed or endorsed by the publisher.
